# Clinical Validation Benchmark Dataset and Expert Performance Baseline for Colorectal Polyp Localization Methods

**DOI:** 10.3390/jimaging9090167

**Published:** 2023-08-22

**Authors:** Luisa F. Sánchez-Peralta, Ben Glover, Cristina L. Saratxaga, Juan Francisco Ortega-Morán, Scarlet Nazarian, Artzai Picón, J. Blas Pagador, Francisco M. Sánchez-Margallo

**Affiliations:** 1 Jesús Usón Minimally Invasive Surgery Centre, E-10071 Cáceres, Spain; lfsanchez@ccmijesususon.com (L.F.S.-P.); jfortega@ccmijesususon.com (J.F.O.-M.); msanchez@ccmijesususon.com (F.M.S.-M.); 2 AI4polypNET Thematic Network, E-08193 Barcelona, Spain; 3 Imperial College London, London SW7 2BU, UK; bglover@ic.ac.uk (B.G.); s.nazarian@imperial.ac.uk (S.N.); 4 TECNALIA, Basque Research and Technology Alliance (BRTA), E-48160 Derio, Spain; cristina.lopez@tecnalia.com (C.L.S.); artzai.picon@tecnalia.com (A.P.); 5 Department of Automatic Control and Systems Engineering, University of the Basque Country, E-48013 Bilbao, Spain; 6 RICORS-TERAV Network, ISCIII, E-28029 Madrid, Spain; 7 Centro de Investigación Biomédica en Red de Enfermedades Cardiovasculares (CIBERCV), Instituto de Salud Carlos III, E-28029 Madrid, Spain

**Keywords:** artificial intelligence, deep learning, clinical validation, survey, polyp detection, polyp localization, colorectal cancer

## Abstract

Colorectal cancer is one of the leading death causes worldwide, but, fortunately, early detection highly increases survival rates, with the adenoma detection rate being one surrogate marker for colonoscopy quality. Artificial intelligence and deep learning methods have been applied with great success to improve polyp detection and localization and, therefore, the adenoma detection rate. In this regard, a comparison with clinical experts is required to prove the added value of the systems. Nevertheless, there is no standardized comparison in a laboratory setting before their clinical validation. The ClinExpPICCOLO comprises 65 unedited endoscopic images that represent the clinical setting. They include white light imaging and narrow band imaging, with one third of the images containing a lesion but, differently to another public datasets, the lesion does not appear well-centered in the image. Together with the dataset, an expert clinical performance baseline has been established with the performance of 146 gastroenterologists, who were required to locate the lesions in the selected images. Results shows statistically significant differences between experience groups. Expert gastroenterologists’ accuracy was 77.74, while sensitivity and specificity were 86.47 and 74.33, respectively. These values can be established as minimum values for a DL method before performing a clinical trial in the hospital setting.

## 1. Introduction

Colorectal cancer (CRC) remains a leading cause of preventable cancer death worldwide [1]. The classical model of CRC tumorigenesis is from sporadic colorectal polyps, which progress over a period of several years before developing malignant potential. This, therefore, allows a window of opportunity for the detection and resection of these premalignant polyps, which has been shown to reduce the incidence of and mortality from CRC [2]. In common with any operator-dependent procedure, colonoscopy and polyp detection has certain limitations. It is understood that small or subtle polyps may be missed at colonoscopy, with an operator-dependent miss-rate for adenomas as high as 26% [3]. In recognition of this, the adenoma detection rate (ADR) has been introduced as a surrogate marker for a thorough endoscopic examination and is one of the key performance indicators for endoscopists. It has been demonstrated that an improved ADR is inversely proportional to post-colonoscopy risk of CRC, with each 1% increase in ADR providing a 3% decrease in the subsequent risk of cancer [4]. There is certainly incentive to maximize polyp detection at the time of endoscopy.

A proportion of the polyps missed during colonoscopy could potentially have been identified if the operator possessed optimal examination skills and polyp recognition capabilities. Evidence for this is shown by studies suggesting that ADR can be increased by improving behavioral and technical skills of the endoscopist [5]. Training programs consisting of hands-on teaching and regular feedback showed positive results in increasing ADR in clinical trials [5,6]. However, the increased ADR in these studies was small and the ability of endoscopists to detect very small, subtle, or flat lesions remains a limiting factor.

Recently, there has been an explosion in the application of artificial intelligence (AI), and its branch deep learning (DL), to provide computer-aided diagnosis (CAD) technologies in medical and health-care diagnostics [7]. The ability of AI, and specifically DL and computer vision approaches, to locate, differentiate, and characterize distinct pathologies can enhance and surpass traditional hand-crafted CAD techniques [8]. Current endoscopic equipment now provides exceptionally high-quality images, with even 1080p HD images or 2K imaging technology, but the presence on non-informative frames due to blurry frames, water frames, and bubble frames is still high [9], which makes it more difficult to distinguish tissues.

Comparison of polyp detection and localization methods is a necessary yet complex process as (1) performance results are highly sensitive to design choices, (2) common metrics do not reflect clinical needs, and (3) comparison across datasets can be misleading [10]. In regard to the latter, it must also be recognized that most datasets present clearly visible polyps, well-centered in the image, although this is not representative of the polyp detection and localization in the clinical setting where lesions often first appear at the edges of the image. This would also be related to point two, as technical metrics do not consider clinical indicators. Thus, clinical needs and casuistry should not be left aside when validating DL methods in a laboratory environment.

Beside comparison between methods, it is also necessary to compare the DL models versus clinicians, to demonstrate the added value of these types of CAD systems in the clinical practice. Many efforts have been made recently in this regard. Li et al. [11] analyzed the performance of AI models for polyp detection and classification in comparison to clinicians, using different imaging modalities. In general, AI systems obtained high sensitivity and moderate specificity for both tasks, similarly to human experts. Nevertheless, none of the works for polyp detection considered endoscopic images and experts, so comparison in this situation is not included. Similarly, Xu et al. [12] do not include any work that compares the DL detection method with clinicians (neither experts nor non-experts) in their meta-analysis. In any case, the ideal validation for any DL model or CAD system would be a randomized control trial (RCT), to prove the actual added value of such systems [13,14,15]. Hassan et al. [16] just included five RCTs in their systematic review, which showed the significant increase in detection when AI systems were used. A small number of RCTs might be due to the limitations for such trials, which might be difficult to overcome for some research groups with limited possibilities, having to face common barriers such as lack of funding, complex regulatory requirements, or inadequate infrastructures [17]. It would be useful to establish a preliminary test that could be used as a prior step to a fully clinical validation and that would allow for a comparison between the DL model and gastroenterologists in a laboratory environment.

The aim of this study is to present the ClinExpPICCOLO dataset, a dataset for the clinical benchmark of DL methods for localization of colorectal polyps when viewing unedited endoscopic images, and to establish an expert clinical performance baseline to use as a comparator when analysing DL methods against clinicians.

## 2. Materials and Methods

### 2.1. ClinExpPICCOLO Dataset

The polyp image library for detection and localization of colorectal polyps was created from the endoscopic videos originating the ‘test’ subset of the PICCOLO dataset [18] to assure subset independence in the event of DL methods trained with this dataset. The PICCOLO dataset in its entirety contains 3433 manually segmented images containing endoscopic images of polyps, captured under WLE and NBI during routine colonoscopy. The ‘test’ set includes 333 images, each of which contains one or more colorectal polyps. These were reviewed individually, and over 100 were selected for inclusion in this study, showing polyps at a variety of sizes, orientations, and distances. For specific details of the acquisition and annotation protocols, the reader is referred to [18].

To supplement these images, representative images that did not contain polyps or showed one or more polyps not centered in the image were selected from the original endoscopic videos. A review group of endoscopists (BG, SN) selected an initial batch of 200 endoscopic images to be used in the study.

To give a comparable benchmark to the ADR of a skilled endoscopist, it was decided that approximately one third of the images should contain a polyp, with the remainder showing no abnormality. The second batch of images were combined with the polyp-containing images from the test set of PICCOLO dataset and reviewed again by the study group. Images that were deemed to be of low quality, or that showed polyps at a high degree of magnification, were removed. Images with a high level of inter-or intra-reviewer disagreement regarding their content were also removed. Lastly, selected images were re-scrutinized to ensure no patient-identifiable data remained within each image. In all, 65 images were selected, 19 of them showing 21 polyps and the remaining 46 with no polyps. Out of the 19 polyp images, 15 were WLE and 4 were NBI, as this modality is preferred for diagnosis rather than for detection of lesions.

All image sizes were either 1920 × 1080 or 854 × 480. In preparation for display to endoscopists, the images were uploaded to a pre-existing digital platform designed for the collection of survey data (Qualtrics, UT, US).

In parallel, the review group of endoscopists agreed on the area of the polyps, for which a binary mask was created using GTCreatorTool [19]. These manually annotated binary masks were used as ground truth.

The ClinExpPICCOLO dataset is publicly available (https://www.ccmijesususon.com/investigacion/clinexppiccolo/, accessed on 17 August 2023). Since the use of the dataset is restricted to research and educative purposes and commercial use is forbidden without prior written permission, a dedicated form to request access must be completed. Clinical metadata is provided as Supplementary Materials in Table S1.

**Figure 1:**
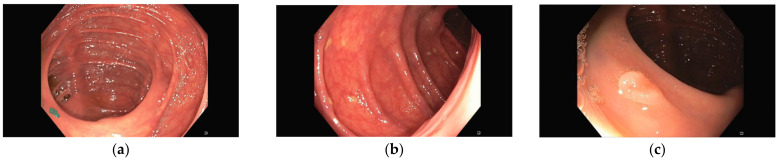

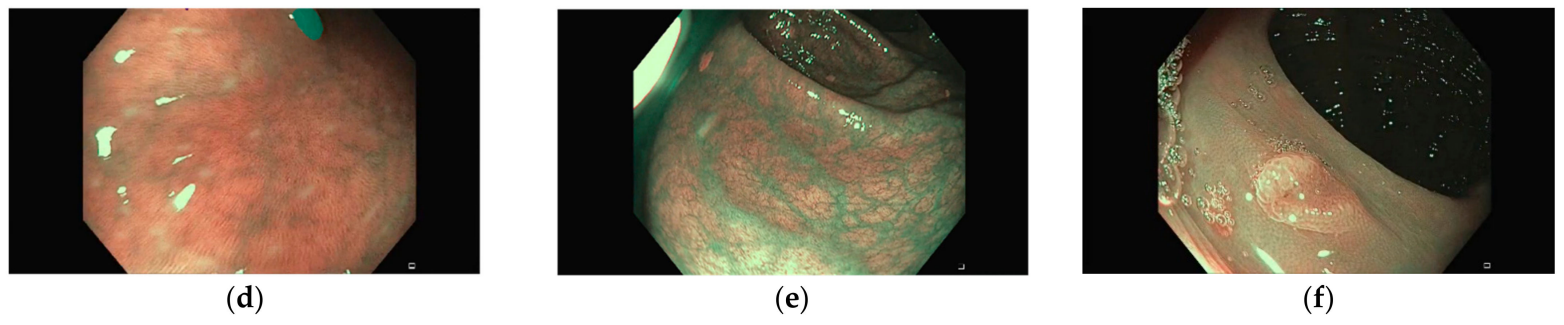
Example images. Upper row images (**a**–**c**) are white light endoscopy (WLE) images, while lower row ones are narrow-band imaging (NBI) images (**d**–**f**). First column (**a**,**d**) corresponds to polyp images (lesions are highlighted for better identification), second column shows non-polyp images (**b**,**e**) and third column (**c**,**f**) are images from the original PICCOLO dataset clearly showing a lesion.

Representative images of the ClinExpPICCOLO dataset are shown in Figure 1. Both WLE and NBI, showing a polyp or not, are included. In comparison to images from the original PICCOLO dataset, images are not focused on the lesions.

### 2.2. Ethics

Ethical approval was obtained for the creation of the PICCOLO dataset [18]. The endoscopic images and videos were collected at Hospital Universitario de Basurto (Bilbao, Spain), under ethical approval of the Ethical Committee of the Basque Country (CEIm-E). Patients provided written informed consent using document PI+CES-BIOEF 2017-03, allowing for use of images in research. As this was an observational survey design involving no identifiable participants, additional ethical approval was not sought for the work detailed in this study.

### 2.3. Deep Learning Models

In this work, four different DL models trained with the PICCOLO dataset have been used (U-Net+VGG16, LinkNet+Densenet121, U-Net+Densenet121, and LinkNet+VGG16) as example. These models are presented in [18] and show the best generalization capabilities among all tested models for polyp segmentation. These models have an encoder–decoder architecture. While the encoder processes the input image and transform it into a feature vector, the decoder reconstructs it into the binary prediction mask, of equal size to the input image.

### 2.4. Clinical Performance Baseline

We recruited participants from local and international professional networks, including digital gastroenterology and endoscopy training networks. A unique URL was sent to potential participants, allowing them to take part in the clinical validation stage.

The images were randomly ordered and presented to participants through the online survey on Qualtrics. This allowed images to be displayed to the survey respondent, and for them to indicate the presence of any polyps in the image by either a mouse click if using a computer, or by a tap on the screen if using a mobile device. Due to the maximum allowed image size, 1920 × 1080 images were automatically resized to 900 × 506, while 854 × 480 remained unresized.

This survey included the instructions to complete it as well as an initial set of demographic questions. Since the survey could be accessed through different devices and, therefore, the displayed image size might vary, the survey also included a question to identify the used device among the following options: desktop computer, laptop computer, tablet computer, and mobile phone.

A limit of three clicks per image was set to avoid unlimited attempts. If a user performed more than three clicks on the same image, the fourth click would replace the first one and so on. In addition, a timer per question was also included. A time limit of 10 s was established. During this time, the user could click polyps on the image and move to the next image, otherwise the survey automatically skipped to the next image when the time ran out. Time and clicks are automatically recoded by Qualtrics. Solutions were not displayed to not bias user behavior depending on whether the click correctly located a polyp or not. Lastly, we also analyzed factors that might influence the results, such as prevalence bias or time pressure.

### 2.5. Calibration Survey

A calibration survey was also created to measure the error of the different input devices. Four black images showing three white crosses each at predefined location were uploaded to Qualtrics. Two images were 1920 × 1080 and the other two were 854 × 480 to account for the image size of endoscopic images. Participants were asked to click in the cross center. Error was measured as the Euclidean distance between each of the three input points and the center of the closest white cross.

### 2.6. Evaluating the Results

Both the clicks from the questionnaire and the predicted binary masks by the DL models were processed to identify whether the polyps were correctly localized or not. To liken the binary masks of the DL models with the clicks provided by gastroenterologists, centroids of each region in the binary masks were calculated and considered as the location points by the DL methods.

Next, the (x,y) coordinates of up to three clicks provided by Qualtrics and those of all centroids were analyzed to obtain the confusion matrix for each user/model and image. Points were labelled as:

- True positive (TP), if the point lay within the area delimitated as polyp in the ground truth. If more than one point was in the polyp area, only one TP was counted. Calibration errors have been considered to correct the location points depending on the input device and image size. Therefore, if the point was within the corresponding error distance from the polyp area indicated in the ground truth, it was also considered as TP;
- False positive (FP), if the point lay outside the area delimitated as polyp in the ground truth.

On the other hand, true negatives (TN) were considered when the ground truth was a black image, and no point was given, while false negatives (FN) were considered when no point lay within the polyp area in the ground truth.

With this convention, a maximum value of 21 TP and 46 TN could be obtained. Based on the elements of the confusion matrix, a set of eight metrics have been calculated:

- Sensitivity

$$Sens=\frac{\mathrm{TP}}{\mathrm{TP}\;+\;\mathrm{FN}}$$

- Specificity

$$Spec=\frac{\mathrm{TN}}{\mathrm{TN}\;+\;\mathrm{FP}}$$

- Accuracy

$$Acc=\frac{\mathrm{TP}\;+\;\mathrm{TN}}{\mathrm{TP}\;+\;\mathrm{TN}\;+\;\mathrm{FN}\;+\;\mathrm{FP}}$$

- Balanced accuracy

$$BAC=\frac{Sens+Spec}{2}$$

- Positive Predictive Value

$$PPV=\frac{\mathrm{TP}}{\mathrm{TP}\;+\;\mathrm{FP}}$$

- Negative Predictive Value

$$NPV=\frac{\mathrm{TN}\;}{\mathrm{TN}\;+\;\mathrm{FN}}$$

- False Negative Rate

$$FNR=\frac{\mathrm{FN}}{\mathrm{FN}\;+\;\mathrm{TP}}$$

- False Positive Rate

$$FPR=\frac{FP}{FP+TN}$$

Furthermore, a simulated ADR was calculated per participant/model. In this case, the original video from which each polyp frame was extracted was considered, as they originated from different patients. In all, there were images from seven unique videos and detection was considered when at least one polyp in the video was identified (i.e., a polyp in any of the images of the video has at least one true positive).

### 2.7. Statistical Analysis

Results of the questionnaires and DL models have been statistically analyzed to identify differences between groups.

Interquartile ranges (IQR) have been calculated for the confusion matrix elements per group of experience, to identify outliers. In this case, outliers are considered those observations 1.5 times the IQR below the first quartile or above the third quartile.

A permutation test implemented in Python 3.7 was used to identify whether differences between groups were statistically significant without assuming any data distribution. One million iterations in the permutation test are calculated and significance is evaluated at *p*-value < 0.05.

## 3. Results and Discussion

### 3.1. Calibration Results

Five answers per device type were collected. Mean values for the distance between the white crosses center and the actual clicks are provided in Table 1. Actual clicks do not show a systematic deviation (offset) in any direction, so values in Table 1 represent random error. Since all images are presented at the same screen size (depending on the device), HD images present a larger error than SD images as they are more reduced in size. As for devices, while desktop and laptop computers have a similar error of two pixels in SD images and four pixels in HD images, mobile devices present a larger error regardless of the image definition. It is worthy to remark that HD images suffer the largest reduction in size when presented in mobile devices, especially mobile phones due to the screen size, so error rockets to 34 pixels. In addition, this is also affected by the input mode, i.e., either a click with a mouse or a tap with the finder on the screen, with the second one being less precise and leading to larger error.

### 3.2. Clinical Performance Baseline Results

In all, 173 answers were obtained, of which 156 were fully completed. The partial answers were not included in the study. Table 2 shows the demographic results.

**Figure 2:**
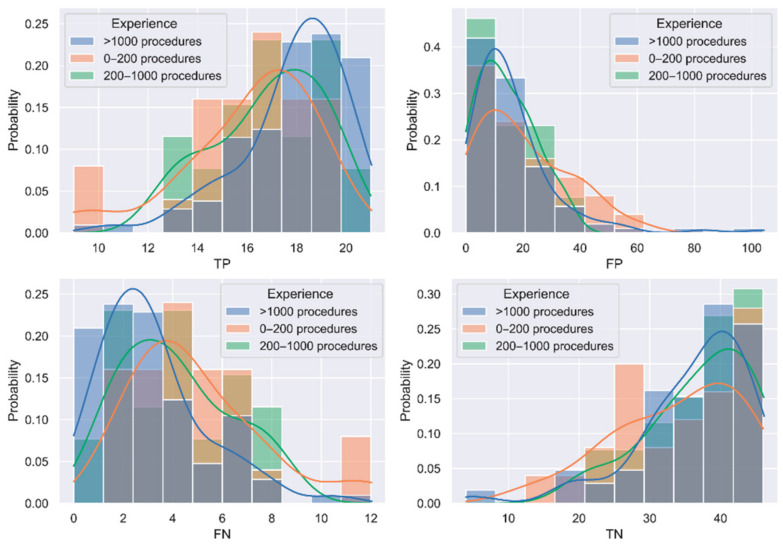
Probability distribution of the confusion matrix elements for each experience group. Outliers are excluded.

In the experienced group (>1000 procedures), ten outliers have been identified and are discarded from the rest of the analysis. Table 3 shows the mean results for the four elements of the confusion matrix and each of the calculated metrics. Experts with over 1000 procedures obtained the best results in all metrics. Figure 2 shows the probability distribution based on the results histogram for the elements of the confusion matrix per experience group. Experts with over 1000 procedures present higher and narrower peaks in the probability distribution in comparison with the other two groups, reflecting a more similar response among all individuals in the group. It is also necessary to remark that variability in the results could also be expected as clinical studies also report great differences in ADR [20,21,22], during training [23], and even during the time of the day [24].

Permutation tests identified significant differences in all pair-wise comparisons of metrics for experts (>1000 procedures) in comparison to the other two groups (*p*-value < 0.05 in all metrics) but not for the less experienced group (<200 procedures) against the intermediate experience group (200–1000 procedures) (*p*-value > 0.25 in all metrics). Therefore, the images included in the dataset show their capacity for construct validity by discriminating experts from the other experience groups. The performance levels obtained by experts should be, thus, the minimum required performance of DL models before moving to the clinical validation trials.

The DL models considered in this study were designed to segment polyps, so despite their generalization capabilities shown in ref. [18], they would not achieve the minimum clinical values for polyp localization in the ClinExpPICCOLO dataset. This also reinforces the need to consider images and metrics similar to those used in clinical practice when training and reporting on the performance of DL models as well as the importance of the task when designing and/or using a particular network architecture.

**Figure 3:**
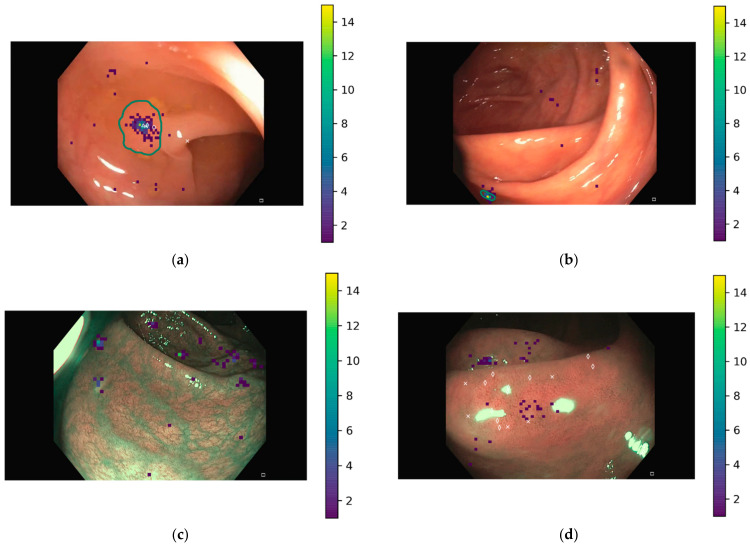
Localization results. Heatmap corresponds to clicks by gastroenterologists while white symbols correspond to DL models. The green line determines the polyp area as indicated by the ground truth mask for images displaying a polyp. (**a**) Correctly located polyp by clinicians and DL models; (**b**) missed polyp by DL models but detected by clinicians; (**c**) false localization by clinicians; (**d**) false localization by clinicians and DL models. (**a**,**b**) are WLE images, while (**c**,**d**) are NBI images.

Localization results were displayed over the endoscopic images to identify causes of FP. Figure 3 shows four examples of images and localization results. The heatmap represents the gastroenterologists’ results, and white symbols corresponds to the DL models. In all, 10 images were identified to present a high number of false positives by the gastroenterologists. To identify the causes, all original videos were reviewed for the strong FP, and it can be confirmed that these are mucosal folds, debris, light reflection, and/or mucosal vascular patterns. Based on the classification by Hassan et al. [25], Table 4 shows a summary of the causes for confusion of these 10 images, indicating also for how many of them the DL models also presented a large number of false positives. Images in which normal mucosa was the cause for the FP, the light reflections caused a polypoid appearance of the mucosa that led to confusion. Other causes of false positives indicated by Hassan et al., either from the bowel wall (such as hemorrhoids) or the bowel content (bubbles), might have a greater presence in full procedures and not in a pool of selected images, where these types of frames have been not considered. Regarding the image light source, eight images were WLE and two were NBI, maintaining the original distribution of polyp images. Therefore, imaging light does not influence the number of FP.

**Figure 4:**
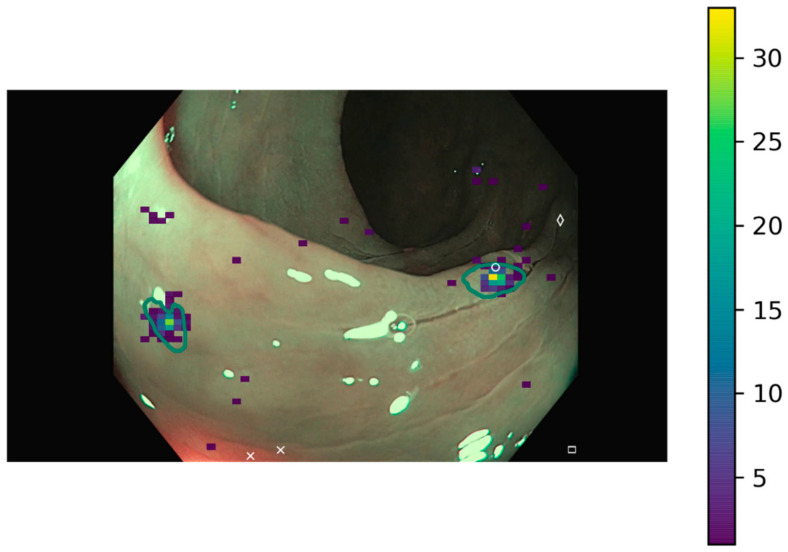
Example image where there are several clicks in the nearby of the ground truth. Heatmap corresponds to clicks by gastroenterologists while white symbols correspond to DL models. The green line determines the polyp area as indicated by the ground truth mask.

It has also been identified in some images that some FP could have been considered as TP if a more flexible threshold would have been established. With the aim of providing an objective framework, only data lying on the lesion area were considered as TP, with a correction based on the calibration error, but it might be necessary to broaden this threshold to account for closer points that could be considered as the lesion is identified (Figure 4). Therefore, the ground truth could be less tight and replaced by bounding boxes or a dilated version of the original binary mask, either by a fixed number of pixels or a certain percentage based on the polyp area. In any of these cases in which the polyp area is modified, the effect of increasing the positive area to assure that clinical relevance is maintained should be fully analyzed, and to ensure that FPs are not artificially increased.

In addition, the type of device was also considered in the analysis. Table 5 shows the aggregated results per device type. As might be expected, the worst results are obtained when a mobile phone is used, because of the smaller screen and lower precision when clicking by tapping on the screen with the finger instead of using a mouse and cursor, even after the error correction.

**Figure 5:**
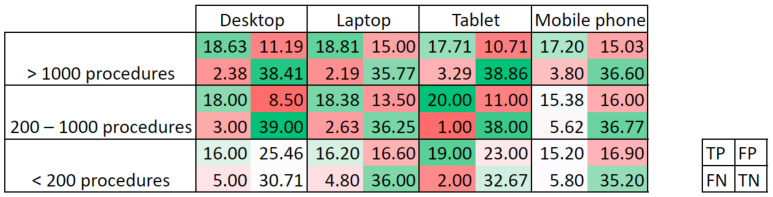
Confusion matrix per experience group and device type. The more intense the color (green for positive elements and red for negative elements), the better the result is.

This trend is even more pronounced if results are split considering the experience groups. Table 6 gathers the number of respondents per experience group and device, while Figure 5 shows the confusion matrix per experience group and device. It can be observed that the trend is to observe more intense colors for more experienced groups and bigger screen size device, not considering the combination of 200–1000 procedures and tablet, since it was only one participant, and it might not be representative of a larger population.

Simulated ADR has also been calculated for the three groups of experience versus type of device (Table 7). Mean ADR is higher when a desktop computer is used, which might be due to both a larger screen but also a larger time for analysis of the images, as it is discussed in the following section.

### 3.3. Clicks and Time Factors

**Figure 6:**
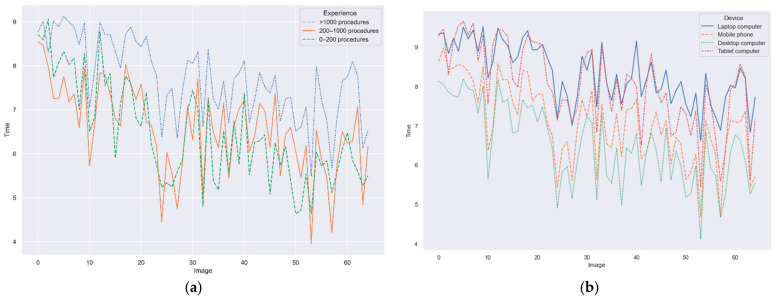
Time to solve each image. (**a**) Per experience group; (**b**) per device type.

The time to solve each image has also been analyzed. As per experience (Figure 6a), more experienced gastroenterologists devote more time than the rest of groups, although the time in each question present a similar trend for each image by the different experience groups, indicating that some images required more time than others to be resolved. In addition, a decreasing tendency in time along the survey can also be observed. This might indicate that users were either more used to the online environment (Qualtrics survey) or willing to finish the study. If device type is considered (Figure 6b), the trend per image is also maintained. Shorter response times are obtained for the desktop computers, which might be justified as these are the most used device in a clinical setting. After that, mobile phones follow. In this case, a smaller screen and tapping on a screen requires shorter response times than navigating in a larger screen of a laptop with a touch pad. Therefore, if further studies along this line shall be performed, the recommended setting would be using a touch screen with a stylus, that would combine the larger screen size of a PC with the ease of use of a tablet.

**Figure 7:**
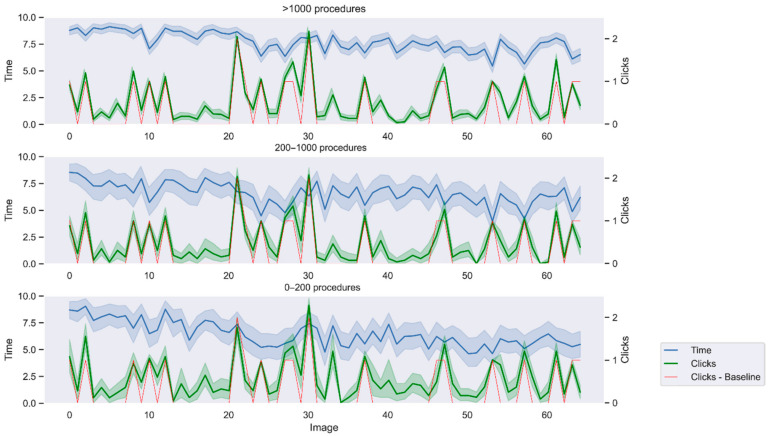
Time and clicks to solve each image. Lines indicate mean value while colored areas indicate the standard deviation. Baseline indicates the number of clicks necessary to identify all polyps in the image, corresponding to the optimal baseline expected.

If time and clicks are considered (Figure 7), experts perform more uniformly than novices in both aspects. Some differences between group can be highlighted. In image 2 showing one polyp (one click required in the optimal baseline), experts adjust to the baseline clicks almost perfectly while less experienced gastroenterologists click more than necessary. On the other hand, images 13 to 20, which show no polyp (zero clicks required in the optimal baseline), present a smaller area under the curve for experts, indicating less false positives than the other groups. Despite time spent in each image decreasing along the survey, it does not imply either more or less clicks, as no bias is observed amongst the different images depending on their position in the survey.

Regarding the number of clicks, results might be related to the prevalence-inducted concept change (PICC). This is the phenomenon in which when the target prevalence is low, more instances are included as target, therefore, increasing its magnitude [26]. It has been determined that feedback influences the decisions and users become conservative if the target prevalence is low [27]. Since feedback is not provided after each click or after each image, gastroenterologists might think that they are being too conservative and counteract this illusory error with more clicks than necessary, therefore, increasing the number of FP, according to the PICC. This effect is less pronounced in more expert gastroenterologists, as expected.

### 3.4. Final Remarks

Comparison of DL methods versus clinical experts have been addressed for the last years to measure the clinical impact of these CAD systems. Systematic reviews and RCTs shows that AI methods can improve clinical performance and ADR [11,12,13,14,15,16]. Nevertheless, setting a pre-clinical validation in which DL methods can be compared to a previously established level of clinical performance is not available yet. The main strength of this work lies on the reproducibility thanks to the open access ClinExpPICCOLO dataset together with the expert clinical performance baseline, which will allow for a fairer comparison of DL methods regarding their performance on images more similar to the clinical ones and also in comparison to experts, suggesting achieving that baseline level before moving to more complex clinical validations. In addition, this work provides results at image level, indicating the precise localization of the polyps, rather than just indicating if an image contains or not a polyp.

On the other hand, this work has also limitations to be acknowledged. Firstly, it is only based on unedited endoscopic images. While these have been selected to represent the clinical setting, and gives gastroenterologists and DL the same information, polyp detection and localization is not performed based on a single frame. Nevertheless, the approach presented in this work could be replicated to create a dataset of videos, although this also presents drawbacks as recordings would be influenced by the expertise of the clinician performing the procedure. On the other hand, this study does not account for fatigue and how it influences on polyp detection and localization. The survey takes as maximum 10 min and 50 s (10 s/image), and although it might be comparable to the mean withdrawal time for a quality colonoscopy set in 7.8 ± 2.4 min [21], it does not account for fatigue during the day [28].

## 4. Conclusions

This work presents the ClinExpPICCOLO dataset, specifically designed for pre-clinical validation of DL models for polyp localization. It presents 65 endoscopic images (19 with polyps and 46 without them) of two different imaging modalities (WLE and NBI). Together with the images, clinical metadata is provided. For the dataset, an expert clinical performance baseline is established by consulting 173 gastroenterologists. Out of them, 146 valid responses are included in the study and used to determine the clinical performance of polyp localization on the ClinExpPICCOLO dataset that can be used as the minimum performance by any DL method before moving to the clinical trials. The number of clicks and time for localization in the survey do not appear to bias results to establish the clinical performance baseline.

## Figures and Tables

**Table 1:** Calibration results. Deviation from the cross center in pixels.

Image Type	Desktop Computer	Laptop Computer	Tablet Computer	Mobile Phone
854 × 480	2.11	2.12	5.60	11.88
1920 × 1080	4.10	4.11	16.18	34.23
All	3.10	3.11	6.84	23.05

**Table 2:** Demographic results.

Item	Values	Number of Answers (%)	Time to Complete the Survey (s)
Experience	<200 procedures	25 (16.03)	567.81 ± 205.68
	200–1000 procedures	26 (16.67)	598.92 ± 249.04
	>1000 procedures	105 (67.30)	701.69 ± 316.38
Device	Desktop computer	44 (28.21)	609.14 ± 381.94
	Laptop computer	42 (26.92)	729.19 ± 201.91
	Tablet	11 (7.05)	735.82 ± 249.15
	Mobile phone	59 (37.82)	642.22 ± 274.60

**Table 3:** Mean value for all metrics for each group. Best value per metric per category is indicated in bold. Ten outliers in the experienced group (>1000 procedures) have been discarded. TP: true positives; TN: true negatives; FP: false positives; FN: false negatives; Sens: sensibility; Spec: specificity; Acc: accuracy; BAC: balanced accuracy; PPV: positive predictive value; NPV: negative predictive value; FNR: false negative rate; FPR: false positive rate.

		# Answers	TP	TN	FP	FN	Sens	Spec	Acc	BAC	PPV	NPV	FNR	FPR
Experience														
	<200 procedures	25	16.08	33.80	19.96	4.92	76.57	65.49	68.16	71.03	52.40	86.88	23.43	34.51
	200–1000 procedures	26	16.88	37.00	13.88	4.12	80.40	73.62	75.46	77.01	60.22	89.97	19.60	26.38
	>1000 procedures	95	**18.16**	**37.15**	**13.41**	**2.84**	**86.47**	**74.33**	**77.74**	**80.40**	**61.54**	**92.87**	**13.53**	**25.67**
Deep learning models														
	Models	4	10.00	36.00	29.25	10.50	48.69	58.14	55.36	53.42	30.39	58.14	51.31	41.86

**Table 4:** Causes of false positives for the 10 images where there is a high number of false positives in the results.

Cause of Confusion	Gastroenterologists	Deep Learning Models
FPs due to artifacts from bowel wall		
Folds	3	1
Normal mucosa	4	1
FPs due to artifacts from bowel content		
Stool	3	0

**Table 5:** Mean value for all metrics for each type of device. Best value per metric is indicated in bold. Outliers have been discarded. TP: true positives; TN: true negatives; FP: false positives; FN: false negatives; Sens: sensibility; Spec: specificity; Acc: accuracy; BAC: balanced accuracy; PPV: positive predictive value; NPV: negative predictive value; FNR: false negative rate; FPR: false positive rate.

		TP	TN	FP	FN	Sens	Spec	Acc	BAC	PPV	NPV	FNR	FPR
Device													
	Desktop computer	18.26	36.58	**14.05**	2.74	86.94	73.65	77.33	80.29	**62.13**	92.79	13.06	26.35
	Laptop computer	**18.38**	35.90	14.90	**2.62**	**87.55**	71.85	76.28	79.70	59.33	**93.23**	**12.45**	28.15
	Mobile phone	16.38	36.38	15.62	4.62	77.99	70.87	72.76	74.43	55.56	88.70	22.01	29.13
	Tablet computer	18.27	**37.09**	14.09	2.73	87.01	**74.16**	**77.56**	**80.59**	61.81	93.17	12.99	**25.84**

**Table 6:** Number of respondents per experience group and device. Outliers have been discarded.

Experience	Device Type			
	Desktop Computer	Laptop Computer	Tablet	Mobile Phone
<200 procedures	32	26	7	30
200–1000 procedures	4	8	1	13
>1000 procedures	7	5	3	10

**Table 7:** Simulated ADR. Outliers have been discarded.

Experience	Device Type				
	Desktop Computer	Laptop Computer	Tablet	Mobile Phone	Mean
<200 procedures	98.66	98.35	97.14	97.96	98.05
200–1000 procedures	100.00	97.14	94.29	100.00	97.14
>1000 procedures	100.00	98.21	96.70	100.00	97.80
Mean	99.00	98.17	96.50	98.70	97.85

## Data Availability

The ClinExpPICCOLO dataset is publicly available (https://www.ccmijesususon.com/investigacion/clinexppiccolo/, accessed on 17 August 2023). Since the use of the dataset is restricted to research and educative purposes and commercial use is forbidden without prior written permission, a dedicated form to request access must be completed.

## Supplementary Materials

The following supporting information can be downloaded at: https://www.mdpi.com/article/10.3390/jimaging9090167/s1, Table S1. Images metadata.
